# High Entropy Oxide Phases with Perovskite Structure

**DOI:** 10.3390/nano10020268

**Published:** 2020-02-05

**Authors:** Denis A. Vinnik, Evgeny A. Trofimov, Vladimir E. Zhivulin, Svetlana A. Gudkova, Olga V. Zaitseva, Dmitry A. Zherebtsov, Andrey Yu. Starikov, Darya P. Sherstyuk, Abdulkarim A. Amirov, Alexandr V. Kalgin, Sergey V. Trukhanov, Fedor V. Podgornov

**Affiliations:** 1 Material science and physics&chemistry of materials, South Ural State University (National Research University), 454080 Chelyabinsk, Russia or sv_truhanov@mail.ru (S.V.T.); 2 Moscow Institute of Physics and Technology (State University), 141701 Dolgoprudny, Russia; 3 Immanuel Kant Baltic Federal University, 236041 Kaliningrad, Russia; amiroff_a@mail.ru; 4 Amirkhanov Institute of Physics Daghestan Scientific Center, Russian Academy of Sciences, 360015 Makhachkala, Russia; 5 Voronezh State Technical University, 394000 Voronezh, Russia; 6 Voronezh State University, 394036 Voronezh, Russia; 7 National University of Science and Technology “MISIS”, 119049 Moscow, Russia; 8 Scientific and Practical Materials Research Centre of NAS of Belarus, 220072 Minsk, Belarus

**Keywords:** phase equilibria, multicomponent oxides, high entropy phases, perovskite, solid-phase sintering

## Abstract

The possibility of the formation of high entropy single-phase perovskites using solid-state sintering was investigated. The BaO–SrO–CaO–MgO–PbO–TiO_2_, BaO–SrO–CaO–MgO–PbO–Fe_2_O_3_ and Na_2_O–K_2_O–CaO–La_2_O_3_–Ce_2_O_3_–TiO_2_ oxide systems were investigated. The optimal synthesis temperature is found between 1150 and 1400 °C, at which the microcrystalline single phase with perovskite structure was produced. The morphology, chemical composition, crystal parameters and dielectric properties were studied and compared with that of pure BaTiO_3_. According to the EDX data, the single-phase product has a formula of Na_0.30_K_0.07_Ca_0.24_La_0.18_Ce_0.21_TiO_3_ and a cubic structure.

## 1. Introduction

The synthesis of high entropy ceramic materials, primarily oxide phases and the study of their properties, is a direction that has been actively developed in recent years. To date, the literature presents the results of properties studies of various oxide high entropy systems [1,2,3,4,5,6,7,8,9,10,11,12,13,14,15] as well as attempts of generalizing the experience of producing such phases [2,3,4,5,6].

Early work in this direction began with the study of rock salt type oxide systems formed only by divalent metals like Mg, Co, Ni, Cu, Zn [8,11,12] or only rare earth elements (Ce, Gd, La, Nd, Pr, Sm, Y) [13,14]. In [15], a high-entropy fluorite type structure was obtained in a system formed by CeO_2_, ZrO_2_, HfO_2_, TiO_2_ and SnO_2_. Later was appeared works devoted to the high entropy oxide systems with a more complex structure. In [16] formation of a multicomponent oxide phase with a spinel structure was reported. Our group initiated studies on high entropy phases with magnetoplumbite structure [17,18,19].

The authors of [20,21,22,23,24] presented high entropy phases with a perovskite structure. In [20], homogeneous high entropy perovskites were obtained in the systems: Sr(Zr_0.2_Sn_0.2_Ti_0.2_Hf_0.2_Mn_0.2_)O_3_, Sr(Zr_0.2_Sn_0.2_Ti_0.2_Hf_0.2_Nb_0.2_)O_3_, Ba(Zr_0.2_Sn_0.2_Ti_0.2_Hf_0.2_Ce_0.2_)O_3_, Ba(Zr_0.2_Sn_0.2_Ti_0.2_Hf_0.2_Y_0.2_)O_(3−*x*)_, Ba(Zr_0.2_Sn_0.2_Ti_0.2_Hf_0.2_Nb_0.2_)O_3_ and (Sr_0.5_Ba_0.5_)(Zr_0.2_Sn_0.2_Ti_0.2_Hf_0.2_Nb_0.2_)O_3_. Analyzing the results, the authors, first of all, compare how much the Goldschmidt rule (connecting the isomorphism with the difference in the ionic radii) allows us to predict the possibility of the system stabilization. The authors of [21] studied the effect of high entropy on the stabilization of a (Gd_0.2_La_0.2_Nd_0.2_Sm_0.2_Y_0.2_)(Co_0.2_Cr_0.2_Fe_0.2_Mn_0.2_Ni_0.2_)O_3_ system. The experiments made it possible to explain the reliance on the configurational entropy of mixing of the system. The authors of [22] obtained Sr((Zr_0.94_Y_0.06_)_0.2_Sn_0.2_Ti_0.2_Hf_0.2_Mn_0.2_)O_3−*x*_ perovskite phase using reactive spark plasma sintering. In [23], the high entropy perovskite film Ba(Zr_0.2_Sn_0.2_Ti_0.2_Hf_0.2_Nb_0.2_)O_3_ was obtained by pulsed laser deposition on SrTiO_3_ and MgO substrates. Finally, in [24], the general problems of the thermodynamic description and modeling of high entropy ceramics were considered. A number of approaches were illustrated using the LaMnO_3±δ_ perovskite as an example.

The present work is aimed at studying the possibility of obtaining high entropy perovskite phases in the BaO–SrO–CaO–MgO–PbO–TiO_2_, BaO–SrO–CaO–MgO–PbO–Fe_2_O_3_ and Na_2_O–K_2_O–CaO–La_2_O_3_–Ce_2_O_3_–TiO_2_ systems. It was assumed that the results of the study will allow us to point out the contribution of heterovalent isomorphism (within A sublattice of perovskite ABO_3_) to the stabilization of high entropy perovskites.

## 2. Materials and Methods

The 99.0%–99.5% pure oxides and carbonates were used as the initial components for the synthesis: BaCO_3_, SrCO_3_, CaO, MgO, PbO, Na_2_CO_3_, K_2_CO_3_, La_2_O_3_, Ce_2_O_3_, Fe_2_O_3_, TiO_2_. The three batch compositions (Table 1) were targeted formulas:

1. (Ba_0.2_Sr_0.2_Ca_0.2_Mg_0.2_Pb_0.2_)TiO_3_;
2. (Ba_0.2_Sr_0.2_Ca_0.2_Mg_0.2_Pb_0.2_)FeO_3_;
3. (Na_0.2_K_0.2_Ca_0.2_La_0.2_Ce_0.2_)TiO_3_.

The thoroughly mixed and ground powders were pressed into pellets. The laboratory resistance furnace with an air atmosphere was used for sintering. The samples were kept in the furnace with the predetermined temperatures for 5 h.

The sintering temperatures optimization was the goal of preliminary experiments. During that stage, the samples were sintered at temperatures from 1000 to 1400 °C with an interval of 50 °C. Then the samples were investigated using the scanning electron microscope Jeol JSM7001F (Jeol, Tokyo, Japan). The criterion for optimal temperature was the size and morphology of the formed crystals. It was necessary to select a temperature that, on the one hand, would ensure the well-shaped perovskite crystals formation and on the other hand this temperature should not lead to the crystal melting.

According to the preliminary experiments the optimal temperature for the first and third composition is 1400 °C, and the second composition it is 1150 °C. Above these temperatures, samples start melting. Since the first and second sample has the only difference in Ti to Fe substitution, it is matter of iron to depress the melting point of a material. At the end of the heat treatment, the samples were cooled, after which the pellets were examined. The Oxford INCA X-max 80 X-ray spectrometer (Oxford, High Wycomb, USA) was used for elemental analysis. The structure was investigated using a Rigaku Ultima IV powder X-ray diffractometer (Jeol, Tokyo, Japan).

For dielectric measurements, the samples were polished to form of thin disks with smooth and parallel surfaces and thicknesses about 0.5 mm. The electric contacts were made from silver adhesive and covered on the top and bottom surfaces of samples and then annealed at 120 °C for 30 min.

The low-field dielectric measurements (real part of permittivity and the loss angle tangent (tan δ)) were carried out at fixed frequencies from 25 Hz to 1 MHz over the temperature range from 20 °C to 600 °C using an immittance meter meter (E7-20 type) (OJSC «MNIPI», Minsk, Belarus). The measurements were conducted in air with rate of 2 °C/min in the heating protocol.

The permittivity as function of temperature were calculated using Equation (1):

(1) $$\varepsilon \left(T\right)=\frac{4dC\left(T\right)}{\varepsilon_{0}\pi D^{2}},$$

from well-known equation for thin capacitor

(2) $$C=\frac{\varepsilon \varepsilon_{0\;}S}{d},$$

where the *S*-area of capacitor’s disc plate with diameter *D* ($S=\frac{\pi *D^{2}}{4})$, *d*—thickness of capacitor, *ε_0_*—electric constant (8.85 × 10^−12^ F/m).

**Figure 1:**
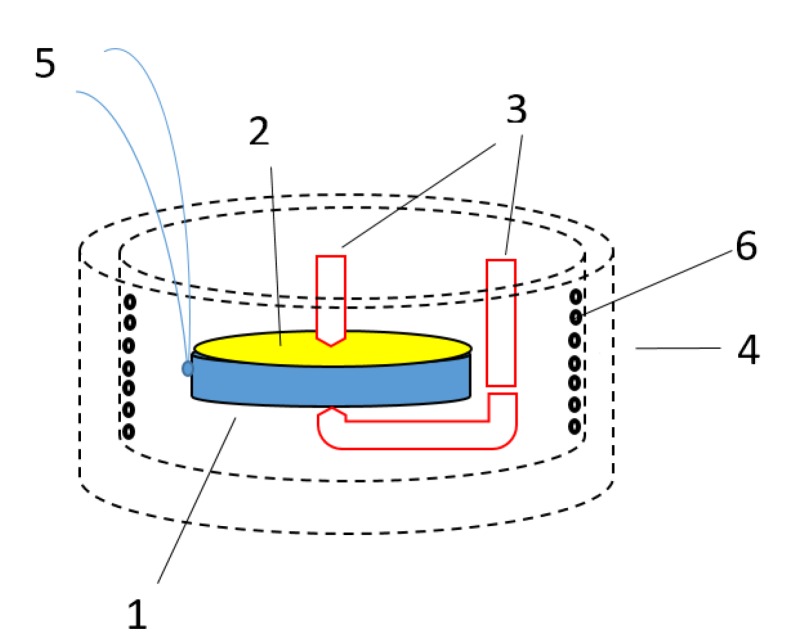
Scheme of dielectric measurements. 1—sample, 2—silver electric contact, 3—connected to LCR-meter electrodes, 4—thermos insulated camera, 5—thermocouple, 6—heater.

The scheme of dielectric measurements shown in Figure 1. The sample (1) in the form of a thin-disc capacitor with silver paste contacts (2) was clamped between special electrodes (3) and connected to LCR-meter. The sample was placed into thermo-insulated adiabatic chamber (4). The temperature of the sample was controlled by K type thermocouple (5) and adjusted by the heater (6). The accuracy of temperature measurements was 0.5 K.

## 3. Results

This section provides a description of the experimental results of the morphology, chemical composition, crystal structure, dielectric properties investigation.

### 3.1. Morphology and Chemical Composition

**Figure 2:**
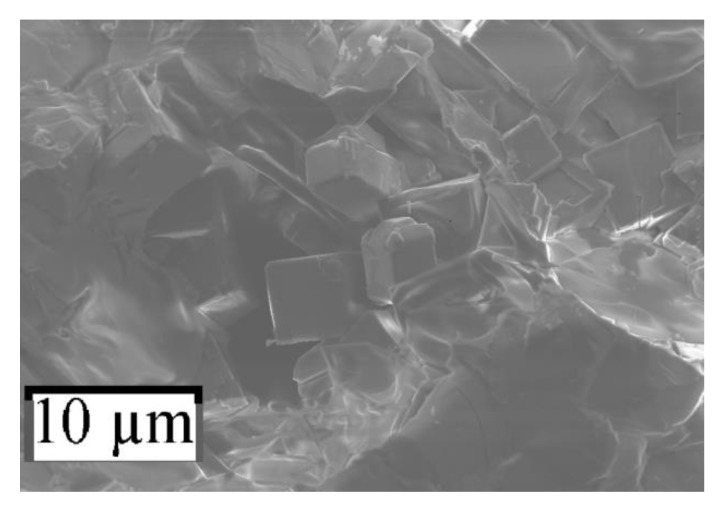
SEM image of sample *№* 1.

**Figure 3:**
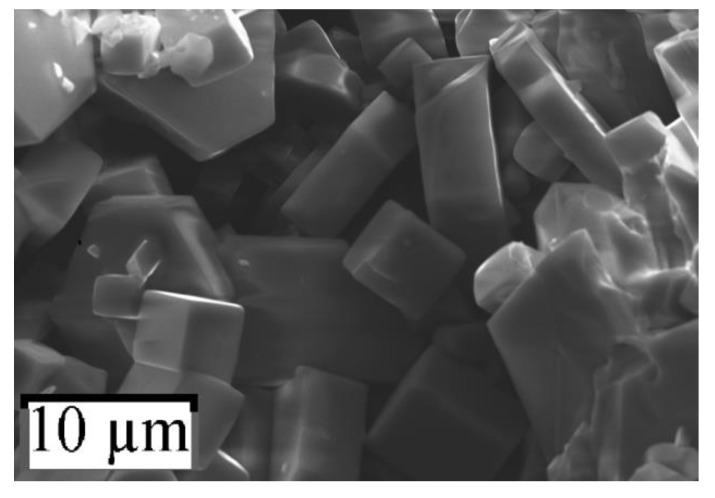
SEM image of sample *№* 2.

**Figure 4:**
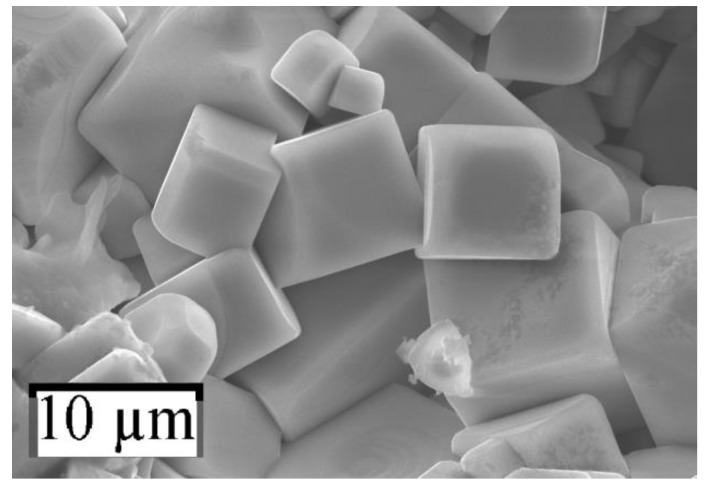
SEM image of sample *№* 3.

The perovskite crystals were formed as cubes of μm size on a free surface of pellets (Figure 2, Figure 3 and Figure 4).

### 3.2. Crystal Structure

**Figure 5:**
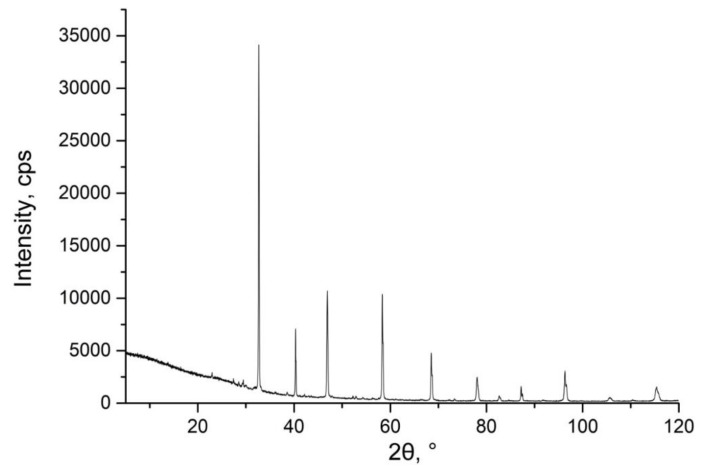
Experimental powder X-ray diffraction pattern of Na_0.30_K_0.07_Ca_0.24_La_0.18_Ce_0.21_TiO_3_ sample.

The powder X-ray diffraction data reveals that samples 1 and 2 contained, besides perovskite, one or more extra phases that made it hard to refer to a certain structure. The composition of some of the extra phases was estimated from EDX of its crystals (Table 2). The Na_0.30_K_0.07_Ca_0.24_La_0.18_Ce_0.21_TiO_3_ diffraction pattern (Figure 5) could be indexed in cubic unit cell with *a* = 3,8650(5) Å. Powder diffractogram confirms high phase purity of this material. The very weak extra reflections at 26–31 °C 2 *θ* belong to 0.5–1.5 mass % of unreacted oxides: La_2_O_3_, CeO_2_ and TiO_2_. The presence of minute amounts of these oxides does not much affect the specific dielectric properties of the main material.

### 3.3. Dielectric Properies

**Figure 6:**
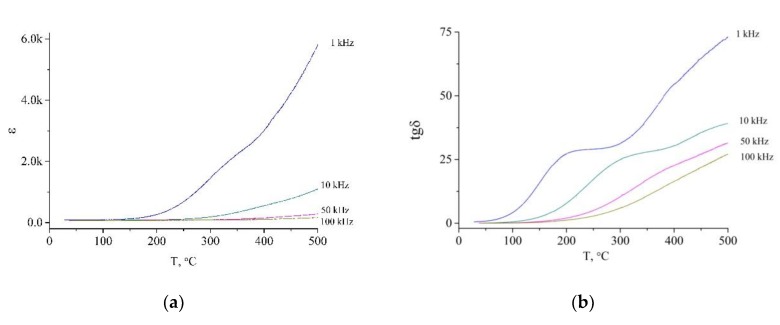
Dielectric parameters of Na_0.30_K_0.07_Ca_0.24_La_0.18_Ce_0.21_TiO_3_: (**a**) real part of dielectric permittivity, (**b**) loss angle tangent.

**Figure 7:**
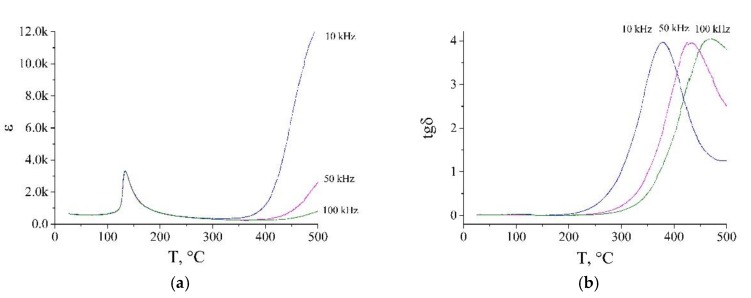
Dielectric parameters of BaTiO_3_: (**a**) real part of dielectric permittivity, (**b**) loss angle tangent.

The real part of dielectric permittivity and loss angle tangent of Na_0.30_K_0.07_Ca_0.24_La_0.18_Ce_0.21_TiO_3_ as well as of pure BaTiO_3_ are demonstrated in Figure 6 and Figure 7 respectively. As one can see, the loss angle tangent of BaTiO_3_ has significantly lower values comparing to Na_0.30_K_0.07_Ca_0.24_La_0.18_Ce_0.21_TiO_3_ at all frequencies within the investigated temperature range. The local maxima of tan δ of the high entropy sample are shifted to the low-temperature range with respect to those of BaTiO_3_ practically at all frequencies of probing voltage. However, there is a significant difference in the thermal behavior of tan δ of Na_0.30_K_0.07_Ca_0.24_La_0.18_Ce_0.21_TiO_3_ and BaTiO_3_. First, it is the growth of the loss angle tangent above 300 °C. At the same time, BaTiO_3_ has the opposite behavior, tan δ vs. T curve demonstrates a single maximum at all frequencies of the probing voltage.

As follows from Figure 6a and Figure 7a, the dependencies of the real part of the permittivity on the temperature of Na_0.30_K_0.07_Ca_0.24_La_0.18_Ce_0.21_TiO_3_ and BaTiO_3_ demonstrate remarkably different behavior. At T ≈ 120 °C, the dielectric permittivity of barium titanate has a peak at all frequencies of the probing voltage. It is explained by the phase transition of BaTiO_3_ from the tetragonal phase to the cubic one. It is worth to mention, that such phase transition is absent in high entropy samples. Indeed, the PXRD pattern at 20 °C is presented only by reflections of the cubic phase. One can speculate on the broadening of temperature stability interval of cubic perovskite due to intensive doping.

With growing temperature, the dielectric permittivity grows for all frequencies for both samples. For Na_0.30_K_0.07_Ca_0.24_La_0.18_Ce_0.21_TiO_3_, its growth begins at significantly lower temperatures (from 200 °C to 300 °C) in comparison with BaTiO_3_ (≈400 °C). This growth is more pronounced for low frequencies, similarly to BaTiO_3_. This effect, as well as thermal behavior of loss angle tangent, can be explained by the relaxation of interfacial polarization (grain-grain boundary). In the case of BaTiO_3_, we can see only one peak in thermal dependence of the loss angle tangent. However, the high entropy sample has several different types of grains therefore it tan *δ* vs *T* curves have overlapping peaks resulting in one growing curve. This approach is also supported by the fact that the *ε*(*T*) is more pronounced at low frequencies (1–10 kHz), which are typical for the interfacial polarization.

## 4. Conclusions

A high entropy single-phase product with a perovskite structure was obtained in the present work. The calculated formula of the substituted phase was Na_0.30_K_0.07_Ca_0.24_La_0.18_Ce_0.21_TiO_3_. From the PXRD data, it was concluded that the high entropy sample was the single perovskite phase. The electrodynamic investigation was performed the morphology, chemical composition, crystal structure, and dielectric properties were studied and compared with pure barium titanate BaTiO_3_ matrix. The polysubstituted high entropy single-phase product contains five doping elements at a high concentration level. The dielectric measurements were performed. The significant influence of high entropy composition on the microcrystals properties was observed. Due to these results, this material can be used in the electric device’s design.

## Figures and Tables

**Table 1:** The batch compositions for synthesis, wt. %.

№	TiO_2_	Fe_2_O_3_	BaCO_3_	SrCO_3_	CaO	MgO	PbO	Na_2_CO_3_	K_2_CO_3_	La_2_O_3_	Ce_2_O_3_
1	37.535	–	18.549	13.877	5.271	3.788	20.98	–	–	–	–
2	–	37.529	18.551	13.878	5.272	3.789	20.982	–	–	–	–
3	44.148	–	–	–	6.2	–	–	5.859	7.64	18.01	18.144

**Table 2:** The phase composition of samples.

№	The Indexed Phases		
1	Ba_0.14_Sr_0.60_Ca_0.48_Mg_0.09_Pb_0.01_TiO_3_	Ba_4_Ti_11_O_26_	Ba_1.2_Ti_6.8_Mg_1.2_O_16_
2	Ba_0.16_Sr_0.43_Ca_0.37_Mg_0.03_Pb_0.08_FeO_3_	BaSrFe_4_O_8_	-
3	Na_0.30_K_0.07_Ca_0.24_La_0.18_Ce_0.21_TiO_3_	-	-
